# Correction: Influence of Cu doping on the visible-light-induced photocatalytic activity of InVO_4_

**DOI:** 10.1039/d0ra90108c

**Published:** 2020-10-13

**Authors:** Natda Wetchakun, Pimonrat Wanwaen, Sukon Phanichphant, Khatcharin Wetchakun

**Affiliations:** Department of Physics and Materials Science, Faculty of Science, Chiang Mai University Chiang Mai 50200 Thailand natda_we@yahoo.com; Materials Science Research Center, Faculty of Science, Chiang Mai University Chiang Mai 50200 Thailand; Program of Physics, Faculty of Science, Ubon Ratchathani Rajabhat University Ubon Ratchathani 34000 Thailand

## Abstract

Correction for ‘Influence of Cu doping on the visible-light-induced photocatalytic activity of InVO_4_’ by Natda Wetchakun *et al.*, *RSC Adv.*, 2017, **7**, 13911–13918, DOI: 10.1039/C6RA27138C.

The authors regret errors in Fig. 4, 7, and 9 in the previously published article. The corrections for the errors in the article are described as follows:

(1) The diffuse reflectance spectra of pure InVO_4_ and 1.0 mol% Cu-doped InVO_4_ are shown in Fig. 4. The absorption margin of 1.0 mol% Cu-doped InVO_4_ was shifted to a longer wavelength, indicating a decrease in the band gap with respect to pure InVO_4_. The absorption margins of the pure InVO_4_ and 1.0 mol% Cu-doped InVO_4_ samples were 505 nm and 510 nm, corresponding to band gaps of 2.51 eV and 2.45 eV, respectively (Fig. 4a and b).

**Fig. 4:**
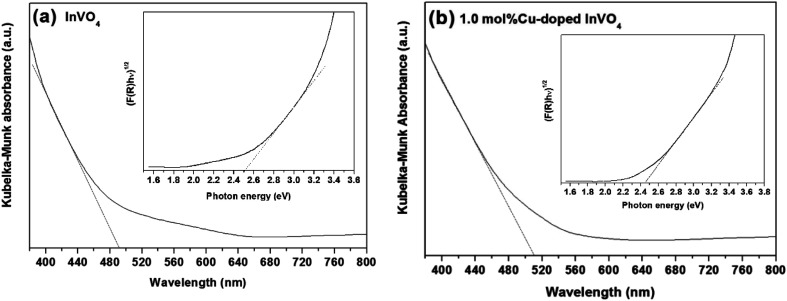
Kubelka–Munk absorbance spectra and band gaps (insets) of the pure InVO_4_ (a) and 1.0 mol% Cu-doped InVO_4_ (b) samples.

(2) The band edge positions of the conduction band (CB) and the valence band (VB) of InVO_4_ can be calculated by the following equation: *E*^0^_CB_ = *χ* − *E*^C^ − 0.5*E*_g_,^1^ where *χ* is the electronegativity of the semiconductor, *E*^C^ is the energy of free electrons on the hydrogen scale of 4.5 eV, *E*_g_ is the band gap of InVO_4_, and the *χ* value of InVO_4_ is 5.74 eV.^2^ The *E*_g_ value of InVO_4_ evaluated from the UV-vis DRS analysis was about 2.51 eV. The valence band energy (*E*_VB_) can be calculated by the following equation:^3^*E*_VB_ = *E*_CB_ + *E*_g_, where *E*_CB_ is the conduction band energy. Based on the equation above, the calculated CB and VB edge potentials of InVO_4_ were −0.02 eV and 2.49 eV, respectively. Now, we are in a position to discuss the photocatalytic mechanism of Cu-doped InVO_4_ for MB degradation (Fig. 7). In the photocatalysis process, when the absorbed photon energy (*hν*) equals or exceeds the band gap, the Cu-doped InVO_4_ generates electron–hole (e^−^/h^+^) pairs. In that case, the generated electrons from the valence band can be transferred to the conduction band of InVO_4_. Since the CB edge potential of InVO_4_ (−0.02 eV) is higher than the standard redox potential, *E*^0^(O_2_/O_2_˙^−^) = −0.33 V *vs.* NHE at pH 7, this suggests that the electrons in the CB of InVO_4_ cannot reduce O_2_ to the superoxide radical ion (O_2_˙^−^). In addition, the VB of InVO_4_ (2.49 eV) is higher than the standard redox potential, *E*^0^(OH^−^/OH˙) = 1.99 V *vs.* NHE at pH 7. This indicates that the photogenerated holes in the valence band of InVO_4_ can oxidize the hydroxyl ion (OH^−^) or water (H_2_O) to form the hydroxyl radical (OH˙).

**Fig. 7:**
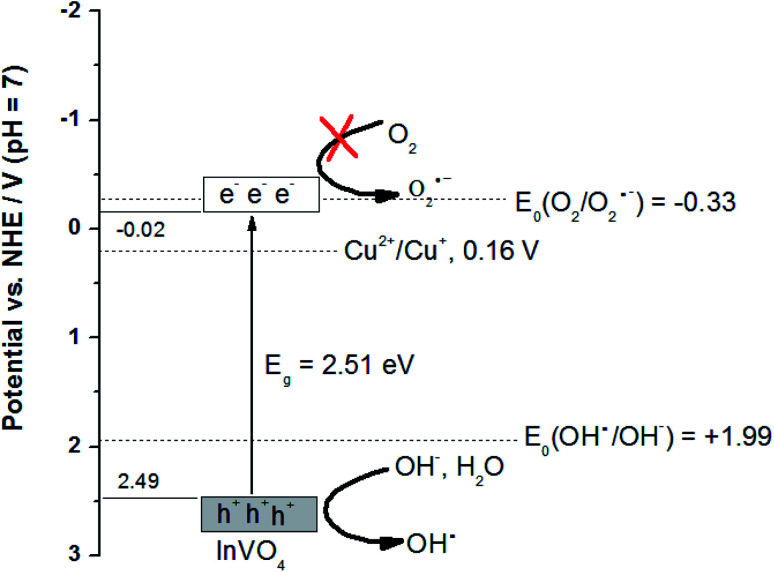
Schematic of the charge migration and separation on Cu-doped InVO_4_.

(3) Due to the contradiction between the scavenging test and the proposed photocatalytic mechanism, Fig. 9 was removed from the original article.

The Royal Society of Chemistry apologises for these errors and any consequent inconvenience to authors and readers.

## Supplementary Material
